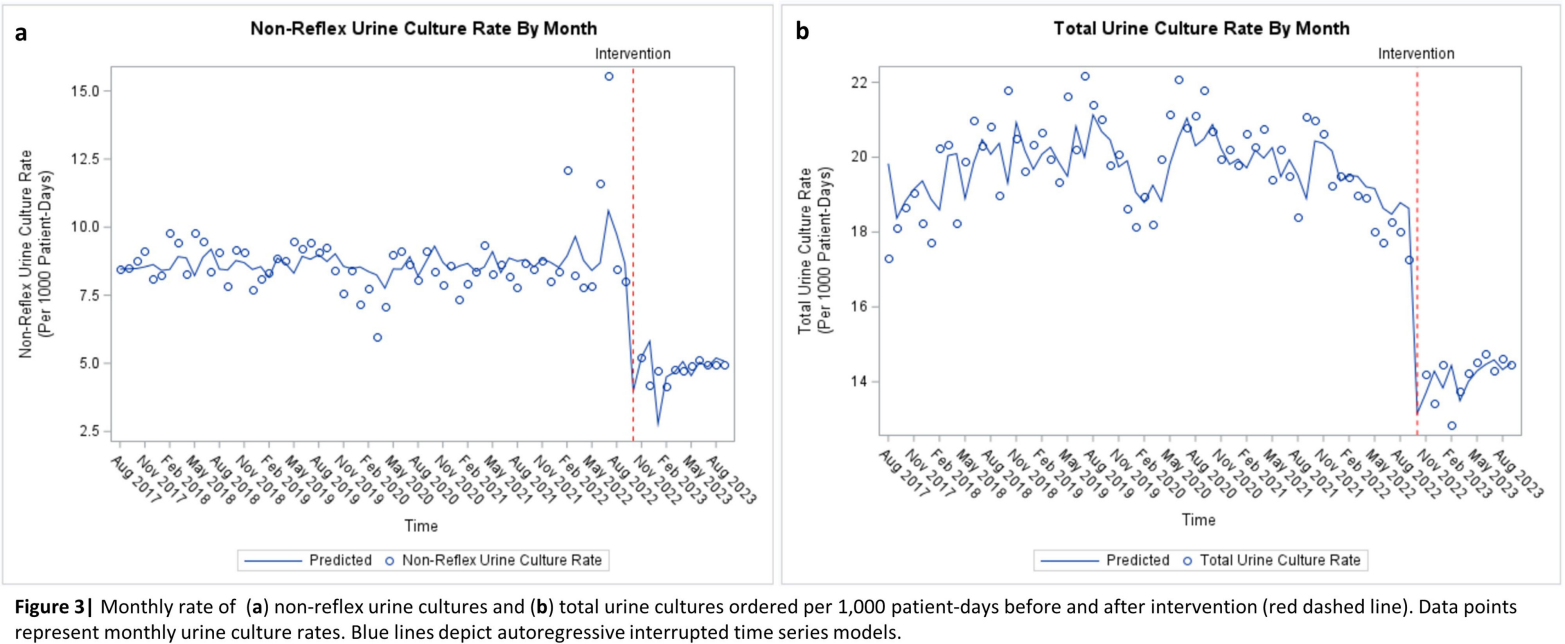# Moving Beyond the Reflex: Effect of a Clinical Decision Support Tool on Urine Culture Ordering Practices

**DOI:** 10.1017/ash.2024.221

**Published:** 2024-09-16

**Authors:** Elizabeth Kim, Julianne Kubes, Shanza Ashraf, Krystle Johnson, Lisa Reif, Kathryn Garcia, Jesse Jacob, Jessica Howard-Anderson

**Affiliations:** Emory University School of Medicine; Emory Healthcare; Emory University Hospital; Emory University

## Abstract

**Background:** Interventions targeting urine culture stewardship can improve diagnostic accuracy for urinary tract infections (UTI) and decrease inappropriate antibiotic treatment of asymptomatic bacteriuria. We aimed to determine if a clinical decision support (CDS) tool which provided guidance on and required documentation of the indications would decrease inappropriately ordered urine cultures in an academic healthcare network that already uses conditional (e.g. reflex) urine testing. **Methods:** In October 2022, four hospitals within one academic healthcare network transitioned to a new electronic health record (EHR). We developed an embedded CDS tool that provided guidance on ordering either a urinalysis (UA) with reflex to urine culture or a non-reflex urine culture (e.g. for pregnant patients) based on the indication for testing (Figure 1). We compared median monthly UA with reflex culture and non-reflex urine culture order rates pre- (8/2017–9/2022) and post- (10/2022–9/2023) intervention using the Wilcoxon rank-sum test. We used interrupted time-series analyses allowing a one-month time window for the intervention effect to assess changes in monthly UA with reflex culture, non-reflex urine culture, and total urine culture order rates associated with the intervention. Using SAS 9.4, we generated Durbin-Watson statistics to assess for autocorrelation and adjusted for this using a stepwise autoregressive model. **Result:** The median monthly UA with reflex culture order rates per 1000 patient-days were similar pre- and post- intervention at 36.7 (interquartile range [IQR]: 31.0–39.7) and 35.4 (IQR: 32.8–37.0), respectively (Figure 2). Non-reflex and total urine culture rates per 1000 patient-days decreased from 8.5 (IQR: 8.1–9.1) to 4.9 (IQR: 4.7–5.1) and from 20.0 (IQR: 18.9–20.7) to 14.4 (IQR: 14.0–14.6) post-intervention, respectively. Interrupted time-series analyses revealed that the intervention was associated with a decrease in the monthly non-reflex urine culture by 4.8 cultures/1000 patient-days (p< 0.001) and in the total urine culture monthly order rates by 5.0 cultures/ 1000 patient-days (p < 0 .001) [Figures 3a and b]. The UA with reflex order rate did not significantly change with the intervention (not pictured). **Conclusion:** In an academic healthcare network that already employed conditional urine testing, the implementation of an EHR-based diagnostic stewardship tool led to additional decreases in both non-reflex and total urine cultures ordered.

**Figure:**
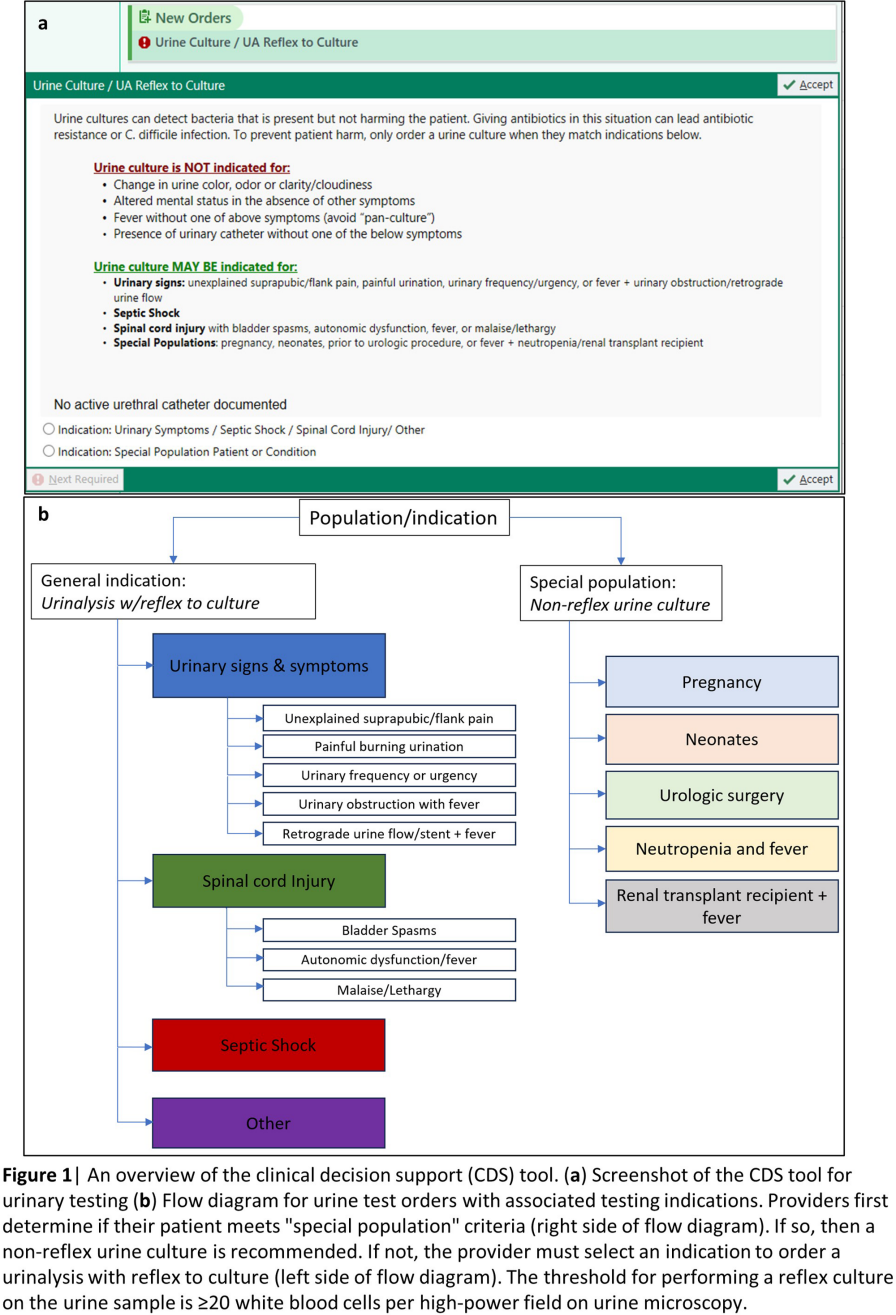

**Figure:**
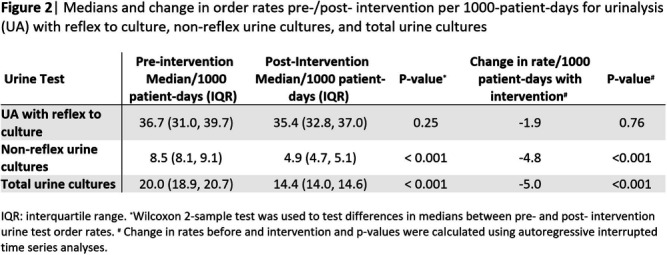

**Figure:**